# A comparison of methods for whole-genome QTL mapping using dense markers in four livestock species

**DOI:** 10.1186/s12711-015-0087-7

**Published:** 2015-02-12

**Authors:** Andres Legarra, Pascal Croiseau, Marie Pierre Sanchez, Simon Teyssèdre, Guillaume Sallé, Sophie Allais, Sébastien Fritz, Carole Rénée Moreno, Anne Ricard, Jean-Michel Elsen

**Affiliations:** INRA, UMR 1388 GenPhySE, BP52627, 31326 Castanet Tolosan, France; INRA, UMR 1313 GABI, Domaine de Vilvert, 78352, Jouy-en-Josas, France; Current address: RAGT-R2n, Le bourg, 12510 Druelle, France; INRA, UMR1282 Infectiologie et Santé Publique, F-37380 Nouzilly, France; Université François Rabelais de Tours, UMR1282 Infectiologie et Santé Publique, 37000 Tours, France; Agrocampus Ouest, UMR1348 Pegase, F-35000 Rennes, France; INRA, UMR1348 Pegase, F-35590 Saint-Gilles, France; Université Européenne de Bretagne, Rennes, France; UNCEIA, Genetics Team, 75595 Paris, France; Recherche et Innovation, IFCE, 61310 Exmes, Paris, France

## Abstract

**Background:**

With dense genotyping, many choices exist for methods to detect quantitative trait loci (QTL) in livestock populations. However, no across-species study has been conducted on the performance of different methods using real data. We compared three methods that correct for relatedness either implicitly or explicitly: linkage and linkage disequilibrium haplotype-based analysis (LDLA), efficient mixed-model association (EMMA) analysis, and Bayesian whole-genome regression (BayesC). We analyzed one chromosome in each of five datasets (dairy cattle, beef cattle, sheep, horses, and pigs) using real genotypes based on dense single nucleotide polymorphisms and phenotypes. The *P* values corrected for multiple testing or Bayes factors greater than 150 were considered to be significant. To complete the real data study, we also simulated quantitative trait loci (QTL) for the same datasets based on the real genotypes. Several scenarios were chosen, with different QTL effects and linkage disequilibrium patterns. A pseudo-null statistical distribution was chosen to make the significance thresholds comparable across methods.

**Results:**

For the real data, the three methods generally agreed within 1 or 2 cM for the locations of QTL regions and disagreed when no signals were significant (e.g. in pigs). For certain datasets, LDLA had more significant signals than EMMA or BayesC, but they were concentrated around the same peaks. Therefore, the three methods detected approximately the same number of QTL regions. For the simulated data, LDLA was slightly less powerful and accurate than either EMMA or BayesC but this depended strongly on how thresholds were set in the simulations.

**Conclusions:**

All three methods performed similarly for real and simulated data. No method was clearly superior across all datasets or for any particular dataset. For computational efficiency and ease of interpretation, EMMA is recommended, but using more than one method is suggested.

**Electronic supplementary material:**

The online version of this article (doi:10.1186/s12711-015-0087-7) contains supplementary material, which is available to authorized users.

## Background

Many methods to detect and localize quantitative trait loci (QTL) in humans and animals are reported in the literature. Current methods work on the basis of identity, either by descent or by state, at either single nucleotide polymorphisms (SNPs) or a short series of SNPs (i.e., a haplotype). Current state-of-the-art methods, especially in human genetics, use a consecutive series of single-marker tests (e.g. association analysis), often corrected by stratification or coancestry [1-4]. The high density of molecular markers ensures that common QTL variants of medium effect will be captured by random linkage disequilibrium of close markers. However, for certain cases, such as causal QTL that are rare variants, simultaneous consideration of consecutive loci (e.g. haplotype association or linkage analysis) can be more powerful or accurate than locus-by-locus analysis (e.g. [5]). In addition, recent research has proposed fitting all markers simultaneously to better model the genetic background and thus improve the analysis [6-8]. Sahana et al. [9] used simulation to compare methods, but comparison of QTL mapping methods using real data has not been reported in animal genetics. The purpose of this study was to compare methods for QTL detection and localization based primarily on real datasets for five traits and four species and complemented with some simulations that mimicked the real data.

Three state-of-the-art methods were compared: LDLA (linkage disequilibrium and linkage analysis), a method that considers haplotypes clustered by approximate identical-by-descent probabilities [10]; EMMA (efficient mixed-model association), a regular single-marker association analysis method for a genome-wide association study (GWAS) but with correction for relatedness in the population [3,11,12]; and BayesC, a Bayesian method that fits effects of all SNPs simultaneously [13]. We examined the following features: number of positive signals, agreement of location of positive signals between the methods, shape of signals, and redundancy of test statistics between neighbouring positions. Data-based simulation was used to characterize method properties (power, accuracy, and false discovery rate) under a pseudo-null hypothesis that considered presence of a QTL effect.

## Methods

Several research projects in the INRA (French National Institute for Agriculture Research) Animal Genetics division have generated a number of datasets that include SNPs and phenotypes. For this study, we considered a variety of distinct experimental designs and species. First, we chose and analyzed chromosomes on which at least one QTL (but not a very large one) had already been detected using the datasets. Then, we considered populations with different family structures: very related (cattle), minimally related but with structure (horses), structured in breeds (pigs), and breed crosses (sheep).

### Description of data

Five datasets, which included four species separately, were examined. One chromosome was analyzed for each dataset.

Cattle data in this study came from on-farm recording. For the horses, the procedures involving animals consisted of radiography and collection of blood samples, which are routinely performed in veterinary practice and are considered noninvasive. All horses were sedated in accordance with clinical guidelines. For the sheep study, all animals were kept indoors, handled with care, and managed as a commercial flock following the INRA ethics policy. At the end of the experimental infection, animals were slaughtered at the INRA-Nouzilly abattoir following the EU rules. For the pig study, animals involved were reared and slaughtered in compliance with national regulations applicable to animal research and commercial slaughtering.

#### Dairy cattle (Bos taurus)

Milk yield (305-day) was examined for a population of 1221 Montbéliarde bulls with a complex pedigree structure that overlapped several generations. Pseudo-performance of bulls was measured by daughter yield deviation [14], which is the average performance of a bull’s daughters corrected for other effects such as herd and genetic merit of dams. The bulls had been genotyped with the Illumina Bovine SNP50 BeadChip. Markers were discarded based on low call rate (<95%), lack of known position on the genome, or very high Mendelian inconsistency (more than 20% parent-offspring discordance), resulting in 43 582 SNPs that were used. Chromosome 1 with 2854 markers was analyzed.

#### Beef cattle (Bos taurus)

Meat tenderness [15] was examined for a population of 936 Blonde d’Aquitaine bulls that had been genotyped with the Illumina Bovine SNP50 BeadChip (43 582 SNPs), filtered as for dairy cattle. Chromosome 7 with 1889 markers was analyzed.

#### Sheep (Ovis aries)

Resistance to nematode infestation measured as faecal egg count after a first infection at 90 days postpartum (FEC12t) [16] was examined for a population of 1067 meat sheep that resulted from a BlackBelly × Romane backcross [17]. Three generations were included: F1, backcross, and backcross × backcross. Animals had been genotyped with the Illumina OvineSNP50 BeadChip (42 469 SNPs, filtered as in [17]). Chromosome 12 with 1424 markers was analyzed.

#### Horse (Equus caballus)

Incidence of hock osteochondrosis [18] was examined for a minimally related but family structured population of 627 French Trotter horses; 102 stallions sired the 525 horses that had been scored for hock osteochondrosis. Animals had been genotyped with the Illumina EquineSNP50 BeadChip (41 249 SNPs, filtered as in [18]). Chromosome 3 with 2267 markers was analyzed.

#### Pig (Sus scrofa)

Length of carcass pre-corrected for breed and other environmental factors [19] was examined for a population of 764 pigs (495 Large White, 129 Landrace, and 140 Piétrain) from 327 sires; 656 pigs had been measured for length of carcass. The entire population had been genotyped with the Illumina PorcineSNP60 DNA Analysis kit (46 865 SNPs, edited as in [19]). Chromosome 17 with 1672 markers was studied.

A brief description of the data is in Table 1.

**Table 1:** **Basic data description by species**

**Descriptor**	**Dairy cattle**	**Beef cattle**	**Sheep**	**Horses**	**Pigs**
Nb of animals	1221	936	1067	627	764
Trait	305-day milk yield (DYD)	meat tenderness	fecal egg count	hock osteochondrosis score	length of carcass
Population structure	large half-sib families, complex pedigree	small half-sib families, complex pedigree	F1, backcross, backcross × backcross	many small families	three breeds
Chr studied	1	7	12	3	17
Nb of chr markers	2854	1889	1424	2267	1672
Length (cM)	161	112	79	119	60
Heritability	0.90	0.20	0.45	0.40	0.30

Nb = number; chr = chromosome; DYD = daughter yield deviation.

### Data analysis

We used three methods to analyse the data: LDLA, EMMA, and BayesC.

#### LDLA

This method was originally developed by Meuwissen et al. [20] and our implementation was as in [10]. Genotypes were first phased based on family and linkage disequilibrium information [21]. Then, haplotypes were defined based on four consecutive (polymorphic) SNPs. Transmission of these haplotypes was traced along the pedigree; if no recombination occurred, haplotypes transmitted from parents to offspring were considered to be identical and carriers of the same allele at the QTL. Founder haplotypes, which cannot have identity-by-descent ascertained, were clustered based on their resemblance, which was based on alikeness in state according to a simple coalescence model. Finally, a variance component model was fitted to the phenotypes for each locus on the chromosome of interest and maximized by restricted maximum likelihood, either with (alternative hypothesis) or without haplotype effects (null hypothesis). The model included a polygenic effect structured according to a pedigree-based relationship matrix: **y** = **1***μ* + **Zu** + **Th** + **e**, where **y** is the vector of phenotypes, **u** and **h** are vectors of polygenic and haplotype effects (with respective incidence matrices **Z** and **T**), respectively, and **e** is a vector of residuals; $Var\left(\mathbf{h}\right)=\mathbf{H}{\sigma}_h^2\ \mathrm{and}\ Var\left(\mathbf{u}\right)=\mathbf{A}{\sigma}_u^2$ are haplotypic and polygenic covariance matrices, with respective variance components ${\sigma}_h^2$ and ${\sigma}_u^2$, with matrix **H** set up according to [10]. A likelihood ratio test comparing the null and alternative hypothesis above was then calculated, and *P* values were computed according to [22]. The process was repeated for each locus on the chromosome of interest; for each locus, new incidence and covariance matrices **T** and **H** were created.

#### EMMA

We used a simple extension of regular regression-based association analysis. The extension fitted a polygenic background via a relationship matrix to deal with structure, as regularly practiced in animal genetics (e.g. [23]) and recently applied in human genetics (e.g. [3]). In our implementation, we used a whole-genome SNP-based relationship matrix (**G**) [24] instead of a pedigree-based relationship matrix. At each marker locus on the chromosome of interest, we fitted the model **y** = **1***μ* + **Zu** + **w***s* + **e**, where **u** is a vector of polygenic effects as in the LDLA model but with $Var\left(\mathbf{u}\right)=\mathbf{G}{\sigma}_u^2$, **w** is a vector of covariates coded as (0,1,2) for each genotype at the analysed SNP, and *s* is the substitution effect of the marker. Effects were estimated using blupf90 [25]. A t-statistic was constructed from the estimate of the substitution effect *s* as *t* = *ŝ*/*s. e*. (*ŝ*) where *s. e*. (*ŝ*) is the standard error of the estimate *ŝ*.

#### BayesC

This method [13] is one of a family of Bayesian methods that were originally conceived for prediction of genetic merit and phenotypes and later extended to map gene locations [6,26]. It has the potential to analyze the whole genome simultaneously. The BayesC model included a set of variable indicators, **d** = {*d*_1_, …, *d*_*n*_}, which indicates if a SNP is (*d*_*i*_ = 1) or is not (*d*_*i*_ = 0) in the model. The phenotypes were thus modelled as **y** = **1***μ* + ∑**w**_*i*_*d*_*i*_*s*_*i*_ + **e**. Solutions of the model were obtained by Gibbs sampling, which provides marginal *a posteriori* inference on **d** in the form of Bayes factors (BF) [27,28]. A key parameter of the model is the *a priori* number of SNPs, which was fixed at Pr(*d*_*i*_ = 1) = 1/1000. Experimentation with 1/100 or 1/10000 did not qualitatively change the results. Gibbs-sampling chains were run for 100 000 iterations using GS3 [29]. All SNPs in the genome were included in the model, although only the SNP estimates for the chromosome of interest were further analyzed. The statistic used was the BF, which corresponds to the increase from prior to posterior probabilities of the SNP being “in” the model [27,30], which in our case can be written as:

$$ B{F}_i=\frac{ \Pr \left({d}_i=0\left|\mathbf{y}\right.\right)/ \Pr \left({d}_i=1\left|\mathbf{y}\right.\right)}{ \Pr \left({d}_i=0\right)/ \Pr \left({d}_i=1\right)}=999\frac{{\widehat{d}}_i}{1-{\widehat{d}}_i}, $$

where ${\widehat{d}}_i$ is the posterior probability of a locus being in the model. For simple models, the BF is numerically identical to the likelihood ratio if *a priori* probabilities are identical [27,30]. Numerically, the scales of the BF and the likelihood ratio are fairly similar [27]. An advantage of BF is that it has a clear interpretation, contrary to the *ad hoc* thresholds used for the likelihood ratio (e.g. [31]), which are not comparable from one study to another.

### QTL detection

We used chromosome-wise Bonferroni correction to infer rejection thresholds for *P* values of LDLA and EMMA, and a BF of 150 for BayesC, because this value was suggested as “very strong evidence” [27] and later used for QTL mapping [32].

Potentially, different methods can reveal different QTL in the same dataset. For instance, for two closely positioned QTL on the same chromosome, classical linkage-based analysis will show a large QTL signal around one location, whereas linkage disequilibrium methods may indicate two separate narrow signals. To determine approximately the ability of methods to disentangle closely positioned QTL, we estimated a measure of redundancy for each method along the chromosome, which was based on the equivalent number of independent tests using Geyer’s [4] effective sample size. This method estimates the number of independent points in a Markov chain process, which in our case consisted of QTL signals along the genome produced by a method.

### Simulations

We simulated data for five scenarios. One hundred replicates were run for each scenario. For each real dataset, we picked at random one or two SNPs as QTL (the same for each replicate within each scenario but not across scenarios) and assigned an effect (the same for each replicate; the effect could be additive or dominant). The effect type, minor allele frequency, and linkage disequilibrium (measured as the correlation between a QTL and the neighbouring marker in strongest linkage disequilibrium with QTL) are in Table 2 for each scenario. The SNP acting as a QTL was then masked. We also simulated a polygenic component based on pedigree relationships, as well as residual deviations, which were added to the QTL genotype effects to simulate phenotypes. Each replicate was analyzed with the three methods (LDLA, EMMA, and BayesC) and for each replicate, the most significant QTL was considered and other significant signals were disregarded. We evaluated three indicators of the quality of QTL mapping: mean squared error in localization (MSE), false discovery rate (FDR), and power. The MSE and power were based on the declared QTL falling within a region around the simulated QTL, and FDR was the number of QTL declared outside the QTL region. Three sizes of QTL regions were examined: ±0.5, ±1, and ±2 cM around the true QTL. The QTL explained 5% of total genetic variance in all scenarios, and the broad-sense heritability of the trait was 0.30.

**Table 2:** **Description of simulated scenarios**

**Scenario**	**Nb of QTL**	**Type of effect**	**MAF**	Linkage disequilibrium*
1	1	Additive	0.3	0.8
2	1	Additive	0.3	0.4
3	1	Additive	0.1	0.8
4	1	Dominant	0.3	0.8
5	2	Additive	0.3	0.8

Nb = number; QTL = quantitative trait loci, MAF = minor allele frequency *correlation between QTL and a close marker.

Statistics for significance decisions (acceptance or rejection of the null hypothesis) generally were not comparable across methods. For example, BF does not have defined type-I and type-II error rates. Therefore, we used the simulations to establish the appropriate rejection thresholds for each statistic (likelihood ratio test for LDLA, t-statistic for EMMA, and BF for BayesC) within each scenario. We examined three thresholds. First, a 5% empirical threshold was computed from the distribution of statistics across all non-QTL positions (those that did not fall in the intervals mentioned above). Because a QTL was included in the simulated data but only non-QTL positions were included in the computation of the statistic, we refer to this as a pseudo-null distribution. Based on 1000 non-QTL positions and 20 simulations, the distribution of the statistic under the pseudo-null hypothesis was described by 20 000 samples. Two more stringent thresholds were also defined. For each of the 100 simulations, the most significant *P* value in the non-QTL regions was retained; then, the 10% and 20% quantiles (computed following [33]) were set as the two thresholds.

After running all simulations, we had five datasets, five scenarios, 100 replicates for each scenario, three methods, three alternative thresholds, and three alternative QTL region sizes to consider as true positives. The average (across 100 replicates) of each QTL quality indicator (MSE, FDR, or power) was calculated, which resulted in 675 values, one for each combination of the above factors.

## Results

### Data analysis

**Figure 1:**
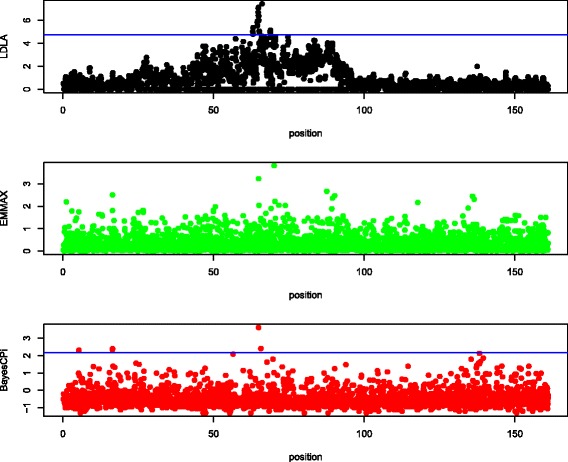
**Manhattan plot of chromosome 1 in dairy cattle.** The y-axis is log_10_(1/*P* value) for LDLA and EMMA and log_10_(Bayes factor) for BayesC; the x-axis is the position along the chromosome in cM; the blue line (if any) is the rejection threshold.

**Figure 2:**
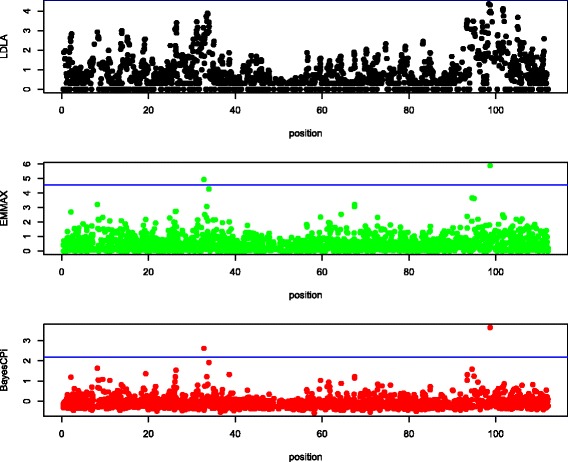
**Manhattan plot of chromosome 7 in beef cattle.** The y-axis is log_10_(1/*P* value) for LDLA and EMMA and log_10_(Bayes Factor) for BayesC; the x-axis is the position along the chromosome in cM; the blue line (if any) is the rejection threshold.

**Figure 3:**
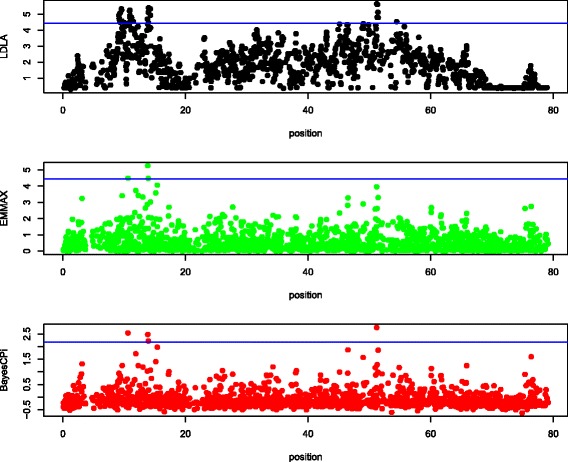
**Manhattan plot of chromosome 12 in sheep.** The y-axis is log_10_(1/*P* value) for LDLA and EMMA and log_10_(Bayes Factor) for BayesC; the x-axis is the position along the chromosome in cM; the blue line (if any) is the rejection threshold.

**Figure 4:**
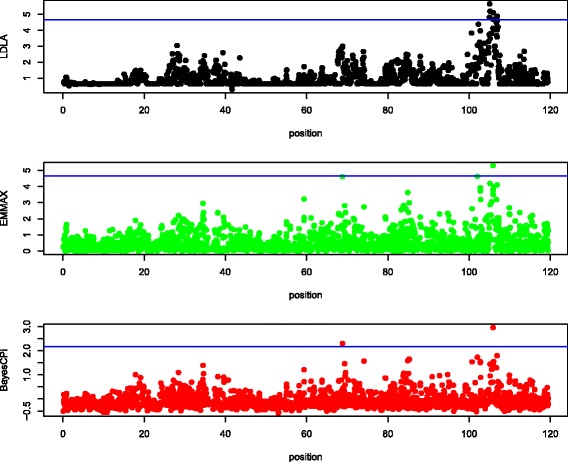
**Manhattan plot of chromosome 3 in horses.** The y-axis is log_10_(1/*P* value) for LDLA and EMMA and log_10_(Bayes Factor) for BayesC; the x-axis is the position along the chromosome in cM; the blue line (if any) is the rejection threshold.

**Figure 5:**
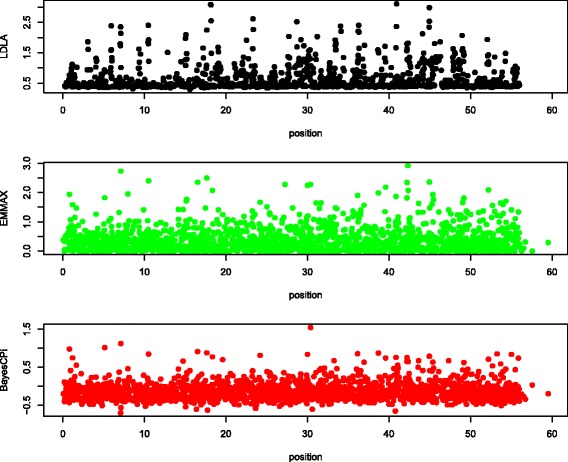
**Manhattan plot of chromosome 17 in pigs.** The y-axis is log_10_(1/*P* value) for LDLA and EMMA and log_10_(Bayes Factor) for BayesC; the x-axis is the position along the chromosome in cM; the blue line (if any) is the rejection threshold.

Manhattan plots of QTL signals for the three detection methods are shown for each data set in Figures 1, 2, 3, 4 and 5.

#### Numbers of positive signals

Table 3 shows the numbers of significant QTL positions detected. These numbers should be regarded with caution because the actual numbers of QTL are not known. Of the three methods, LDLA had the largest number of signals for dairy cattle, horses, and sheep. For beef cattle and pigs, no significant association was found using any of the three detection methods.

**Table 3:** **Numbers of significant positions detected for quantitative trait loci by species and detection method**

**Method**	**Dairy cattle**	**Beef cattle**	**Sheep**	**Horses**	**Pigs**
LDLA*	20	0	29	6	0
EMMA**	0	2	3	1	0
BayesC***	6	2	4	2	0

*statistic = likelihood ratio test corrected by Bonferroni; **statistic = t-statistic corrected by Bonferroni; ***statistic = Bayes factor.

#### Agreement

Because the objective of QTL mapping is not only to detect QTL but to provide locations for further investigation by molecular geneticists, agreement of the location of positive signals between the three detection methods was evaluated. Informal visual agreement between methods is evident from Figures 1, 2, 3, 4 and 5. Additional Tables S1, S2, S3, S4 and S5 [See Additional file 1: Tables S1, S2, S3, S4 and S5] show exact locations and statistics for the two major signals found in each analysis. For example, the dairy cattle data showed a QTL across methods in a window of 2 cM around 65 cM and beef cattle data show agreement between methods of a QTL around 32 and 98 cM, etc. For sheep and beef cattle, the figures show more agreement than the additional tables, because the peaks in agreement may be first or second highest signals in one method but third or fourth highest signals in another. Only the pig dataset did not show clear agreement for detected QTL, but no significant positive signals were found for pigs. We believe that the true signals that were present in these datasets were found by all methods (within a bracket of 1 to 5 cM) and that false signals were random noise and, therefore, without agreement.

#### Redundancy

Estimates of the numbers of independent tests are in Table 4. Redundancy increased with family structure (*i.e.* number of half- and full-sibs within family); e.g. in horses, the large number of small families resulted in low redundancy. In general, LDLA tended to be the most redundant (i.e. a small number of independent tests). Redundancy was less for EMMA and BayesC than for LDLA because (1) haplotypes (or haplotype structure) are highly correlated from one position to the next (but SNP effects are not) and (2) LDLA is partly family (linkage) based (but EMMA and BayesC are not). In addition, BayesC estimators are marginalized over all other loci.

**Table 4:** **Numbers of independent tests by species and detection method**

**Method**	**Dairy**	**Beef**	**Sheep**	**Horses**	**Pigs**
LDLA*	237	183	153	978	380
EMMA**	2323	1082	770	968	1672
BayesC***	2390	1889	1108	1303	1509

*statistic = likelihood ratio test corrected by Bonferroni; **statistic = t-statistic corrected by Bonferroni; ***statistic = Bayes factor.

### Simulations

Quality indicators for QTL detection methods are in Table 5 as least-squares means (estimated marginal means over a balanced design across all other simulation factors [34]). The LDLA method had not only lower power but also higher MSE and FDR. The EMMA and BayesC methods were clearly superior to LDLA in the simulations, regardless of scenario or simulation factor (data not shown).

**Table 5:** **Estimated mean squared errors (MSE), power, and false discovery rates (FDR) of detection methods across simulation factors**

**Method**	**MSE**	**Power**	**FDR**
BayesC*	0.22	0.36	0.007
EMMA**	0.21	0.36	0.006
LDLA***	0.39	0.26	0.017

*statistic = likelihood ratio test corrected by Bonferroni; **statistic = t-statistic corrected by Bonferroni; ***statistic = Bayes factor; standard errors = 0.02 for MSE, 0.005 for power, and 0.0005 for FDR.

## Discussion

Overall, the results were similar with the three QTL detection methods. The Manhattan plots had similar profiles and indicated the same QTL locations for significant (or close to significant) points. None of the methods appeared to produce spurious peaks because of population stratification. All three methods accounted for relationships either through pedigree or genome-wide markers.

Table 3 suggests that LDLA is more powerful than EMMA or BayesC. However, in Figures 1, 2, 3, 4 and 5, some multiple signals obtained with LDLA corresponded to a single peak obtained with EMMA and BayesC, which suggests that many LDLA signals echo a single QTL. This makes sense because the covariance structure for haplotypes changes little from one position to another (as evident in Table 4); i.e. LDLA uses linkage information. All QTL regions that were detected by LDLA were also detected by EMMA and BayesC; thus, LDLA was not more powerful than EMMA or BayesC.

The performance of the methods differed slightly between the real and the simulated data. For example, LDLA produced more positive signals with real data than EMMA and BayesC. In contrast, for simulated data (Table 5), LDLA was the least powerful method. Several explanations for this contradiction are possible. One is that only one QTL was simulated, whereas many potential QTL exist in the real data. Another explanation is that LDLA produces several correlated signals for a single QTL, as discussed previously. Still another explanation is that the nominal threshold used for the real data was too liberal and, therefore, produced false positives. The threshold to be used for the real data is also debatable (e.g. [35]). Although a reasonable consensus exists on how to calculate a threshold under a strict null hypothesis (no QTL on the chromosome), such a hypothesis is nonsensical because biologically there must be many, possibly small QTL on each chromosome. Therefore, a pseudo-null hypothesis was used for our simulations. As an example of the problems caused by the use of rejection thresholds, the two highest (although non-significant) peaks of EMMA in Figure 1 agree with the significant peaks obtained with LDLA and BayesC. In Figure 2, (non-significant) results from LDLA agree with results from the other methods. Therefore, no one method is clearly the most powerful for a given dataset because the ideal rejection threshold depends on the true genetic architecture of a dataset. Thus, a common sense approach is to use several different methods for QTL detection.

For QTL location, the methods were in agreement for the real data. This was as expected because the chromosomes were suspected to contain QTL. For the pig data, no agreement for QTL location was found among the methods, but no signals were significant; using several methods and obtaining no significant signals indicates the absence of detectable QTL for this dataset. However, for the beef cattle data, no significant QTL were found with LDLA but significant QTL were evident when using EMMA and BayesC.

The results from the BayesC and EMMA methods agreed to a very large extent for the real and simulated data because they both model each SNP as having an effect. The complexity of BayesC and lack of a consensual rejection threshold for BayesC might make EMMA preferable. If BayesC is used, Bayesian testing (i.e. using BF) is recommended, since it produced results that are comparable within and across datasets.

Finally, LDLA results in a more correlated structure of signals across the genome. This was evident as less power and accuracy in QTL mapping with simulated data but was not clear in the real data. Therefore, the larger number of independent tests found for EMMA and BayesC does not necessarily imply greater accuracy.

Some discussion about the scope of this work is also needed. Many studies have compared QTL detection methods based on simulations. In those simulations, the QTL location and action are precisely described. However, very little is known about QTL action in real data and, thus, such simulations tend to provide incomplete results because they do not account well for the range of possible gene actions. Phenomena such as self-regulation, epistasis, and pleiotropy are particularly difficult to simulate. Our study, which was based on real data, attempts to address the question of whether analysis of a dataset with another method will provide a different result. We have given some empirical answers to this question.

## Conclusions

All three methods (haplotype-based, association analysis with relationship matrices, and Bayesian analysis) were suitable for QTL detection. Using several methods for the same dataset is recommended. EMMA was easier to use and had slightly higher accuracy and power for both real and simulated data. For QTL with large signals, the methods agreed well. Establishment of significance thresholds is difficult because it is unclear what the null hypothesis should be [35]. The use of nominal *P* values corrected for multiple-testing for the frequentist methods and of BF for the Bayesian method resulted in acceptable statistical properties and led to similar conclusions across methods and datasets.

## Additional file

Additional file 1: Table S1. Dairy cattle: positions of the first and second largest signals for each method. Positions of two largest QTL detection signals. **Table S2.** Beef cattle: positions of the first and second largest signals for each method. Positions of two largest QTL detection signals. **Table S3.** Horses: positions of the first and second largest signals for each method. Positions of two largest QTL detection signals. **Table S4.** Title: Sheep: positions of the first and second largest signals for each method. Positions of two largest QTL detection signals. **Table S5.** Pigs: positions of the first and second largest signals for each method. Positions of two largest QTL detection signals.
