# Supplementary material for: A comparison of methods for whole-genome QTL mapping using dense markers in four livestock species
Source: Genet Sel Evol. 2015 Feb 12;47(1):6. doi: 10.1186/s12711-015-0087-7 (PMC4324410; doi:10.1186/s12711-015-0087-7)
Supplement: Additional file 1: Table S1. — Dairy cattle: positions of the first and second largest signals for each method. Positions of two largest QTL detection signals. Table S2. Beef cattle: positions of the first and second largest signals for each method. Positions of two largest QTL detection signals. Table S3. Horses: positions of the first and second largest signals for each method. Positions of two largest QTL detection signals. Table S4. Title: Sheep: positions of the first and second largest signals for each method. Positions of two largest QTL detection signals. Table S5. Pigs: positions of the first and second largest signals for each method. Positions of two largest QTL detection signals. [file 12711_2015_87_MOESM1_ESM.pdf]

Supplementary material

TABLE S1. Dairy cattle: positions of the first and second largest signals for each method

	First peak		Second peak	
	Position	Log10 (p-value BF)	Position	Log10 (p-value BF)
LDLA	66.09	7.44*	64.90	7.13*
EMMA	70.09	3.81	64.93	3.23
BayesC	64.09	3.62*	65.71	2.42*

*Significant

TABLE S2. Beef cattle: positions of the first and second largest signals for each method

	First peak		Second peak	
	Position	Log10 (p-value BF)	Position	Log10 (p-value BF)
LDLA	98.40	4.37	98.69	4.32
EMMA	98.70	5.89*	32.77	4.93*
BayesC	98.70	3.65*	32.77	2.62*

*Significant

TABLE S3. Horses: positions of the first and second largest signals for each method

	First peak		Second peak	
	Position	Log10 (p-value BF)	Position	Log10 (p-value BF)
LDLA	105.05	5.66*	105.13	5.21*
EMMA	105.88	5.29*	102.03	4.62
BayesC	105.88	2.96*	68.81	2.31*

*Significant

TABLE S4. Sheep: positions of the first and second largest signals for each method

	First peak		Second peak	
	Position	Log10 (p-value BF)	Position	Log10 (p-value BF)
LDLA	51.20	5.66*	51.31	5.62*
EMMA	13.83	5.26*	10.61	4.49*
BayesC	51.19	2.75*	10.61	2.74*

*Significant

TABLE S5. Pigs: positions of the first and second largest signals for each method

	First peak		Second peak	
	Position	Log (p-value BF)	Position	Log (p-value BF)
LDLA	40.93	3.11	18.13	3.09
EMMA	42.32	2.91	7.10	2.73
BayesC	30.38	1.55	7.10	1.13
